# Lactones from Unspecific Peroxygenase-Catalyzed In-Chain Hydroxylation of Saturated Fatty Acids

**DOI:** 10.1021/acs.orglett.3c01601

**Published:** 2023-06-30

**Authors:** Ana C. Ebrecht, Thato M. Mofokeng, Frank Hollmann, Martha S. Smit, Diederik J. Opperman

**Affiliations:** † Department of Microbiology and Biochemistry, University of the Free State, Bloemfontein 9300, South Africa; ‡ Department of Biotechnology, Delft University of Technology, Delft 2629HZ, The Netherlands

## Abstract

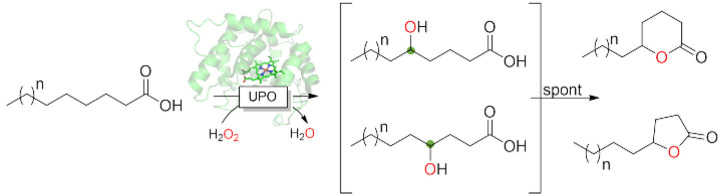

γ- and δ-lactones are valuable flavor and fragrance compounds. Their synthesis depends on the availability of suitable hydroxy fatty acid precursors. Three short unspecific peroxygenases were identified that selectively hydroxylate the C4 and C5 positions of C8–C12 fatty acids to yield after lactonization the corresponding γ- and δ-lactones. A preference for C4 over C5 hydroxylation gave γ-lactones as the major products. Overoxidation of the hydroxy fatty acids was addressed via the reduction of the resulting oxo acids using an alcohol dehydrogenase in a bienzymatic cascade reaction.

Unspecific peroxygenases (UPOs) make up a rapidly growing class of heme-thiolate enzymes used for biocatalytic oxyfunctionalization reactions, including C(sp^3^)–H hydroxylation, C(sp^2^)–C(sp^2^) epoxidation, and aromatic hydroxylation. These hydroxylation reactions are, as with cytochrome P450 monooxygenases (CYPs/P450s), mediated via a high-valence iron(IV) porphyrin-π-cation radical (compound I). Moreover, UPOs can catalyze alcohol oxidation, demethylation, and one-electron oxidation reactions.^1−4^ However, unlike P450s that require molecular oxygen, reducing equivalents from expensive cofactors such as NAD(P)H and additional redox partner proteins to deliver the electrons to the catalytic heme, UPOs require only H_2_O_2_ for heme activation to the reactive compound I species.^5,6^ This simplicity and available enzymatic or electro(chemical) methods for *in situ* production of H_2_O_2_^7^ make UPOs ideally positioned for scalable reactions.^6^

To date, UPOs have been used for the epoxidation of various unsaturated fatty acids^8−10^ and the terminal^11^ and subterminal hydroxylation of saturated fatty acids.^12,13^ These reactions have long been established within the CYP field using CYP153s and CYP52s for terminal hydroxylation and CYP102s (such as P450BM3) for subterminal hydroxylation of fatty acids, fatty alcohols, and *n*-alkanes.^14−16^ More recently, however, the focus has shifted to selective in-chain hydroxylation, specifically hydroxylation at C4 and C5 of saturated fatty acids and fatty alcohols by CYPs. C4 and C5 hydroxy fatty acids can readily be cyclized to γ- and δ-lactones, respectively, whereas the corresponding diols can be lactonized using alcohol dehydrogenases (ADHs).^17,18^ Biotechnological production of γ- and δ-lactones for the flavor and fragrance industry has traditionally depended on the extraction of natural hydroxy fatty acids subjected to repeated cycles of β-oxidation by fungi or yeast. Alternatively, the hydration of unsaturated fatty acids using hydratases and the hydroxylation using lipoxygenases are used to produce the starting hydroxy fatty acids. These biocatalytic oxyfunctionalization and subsequent β-oxidation routes, however, offer an only limited number of possible γ- and δ-lactones.^19^ We, and others, have explored CYPs for the selective in-chain hydroxylation of saturated fatty acids for the production of δ-lactones. CYP505E3 hydroxylates dodecanoic acid or dodecanol at ω-7 (C5) for the production of δ-dodecalactone,^18,20^ and CYP116B46 has been shown to hydroxylate both decanoic and dodecanoic acid at C5 to form the corresponding δ-lactones.^21^ To date, only the lactonization of methyl hexanoate has been achieved with a UPO, through typical subterminal (ω-1) hydroxylation.^22^ Here we report the first UPO-catalyzed lactonization of octanoic, decanoic, and dodecanoic acid to their corresponding γ- and δ-lactones.

A small panel of five short UPOs (Table S1) was screened for the hydroxylation of C8–C12 saturated fatty acids. Four of these UPOs (*Cvi*UPO from *Collariella virescens*, *Dca*UPO from *Daldinia caldariorum*, *Mro*UPO from *Marasmius rotula*, and *Hsp*UPO from *Hypoxylon* sp. EC38) have previously been characterized for *n*-alkane hydroxylation,^11^ epoxidation and allylic hydroxylation of terminal alkenes,^23^ unsaturated fatty acid epoxidation^10,24,25^ and subterminal hydroxylation,^26^ benzylic hydroxylation,^27^ alcohol oxidation,^26,27^ epoxidation,^27^ naphthalene oxidation,^26^ and indole oxidation.^27^ An additional uncharacterized UPO from *Talaromyces rugulosus* (*Tru*UPO), identified through BlastP analysis of the NCBI database, was included. The sequence of *Tru*UPO is <50% identical with those of the four characterized UPOs. All five UPOs were heterologously expressed in *Escherichia coli* without their signal sequences but containing an N terminal hexahistidine tag. The UPOs were purified to near homogeneity (Figure S1a), and their activity was confirmed through the apparent ubiquitous one-electron oxidation of ABTS displayed by UPOs, as well as CO-difference spectra (Figure S1b). The purified UPO is obtained from *E*. *coli* in amounts ranging from 3 to 24 mg/L of culture (Table S2), which is comparable to that previously observed for UPOs expressed in *E*. *coli*(26) but lower than what has been achieved with the engineered long UPO variant, PaDa-1, in *Pichia pastoris*.^28^

Initial screening was performed using 10 mM fatty acid and commercial glucose oxidase (GOx) from *Aspergillus niger* for *in situ* H_2_O_2_ production. Reactions (1 mL) were run with 20 μM UPO for 24 h, and then the mixtures were acidified and extracted. Near complete conversion of octanoic acid was observed with *Dca*UPO, *Hsp*UPO, and *Tru*UPO, with the γ- and δ-lactones as the major products (Figure 1a), resulting from the spontaneous cyclization of the C4 and C5 hydroxy fatty acids (Scheme 1). The intramolecular esterification reaction was not quantitative as small amounts of uncyclized 4- and 5-hydroxy octanoic acid were observed. This is most likely due to the reversible character of the esterification reaction and will be addressed in future studies using tailored reaction media (low water activities) to shift the equilibrium even further. Moreover, the corresponding C4 and C5 oxo acids were also detected due to the overoxidation of their respective hydroxy fatty acids. This overoxidation is not uncommon in UPO-catalyzed reactions^3^ and proceeds via sequential hydroxylation resulting in a *gem*-diol to form the ketone.

**Scheme 1:**
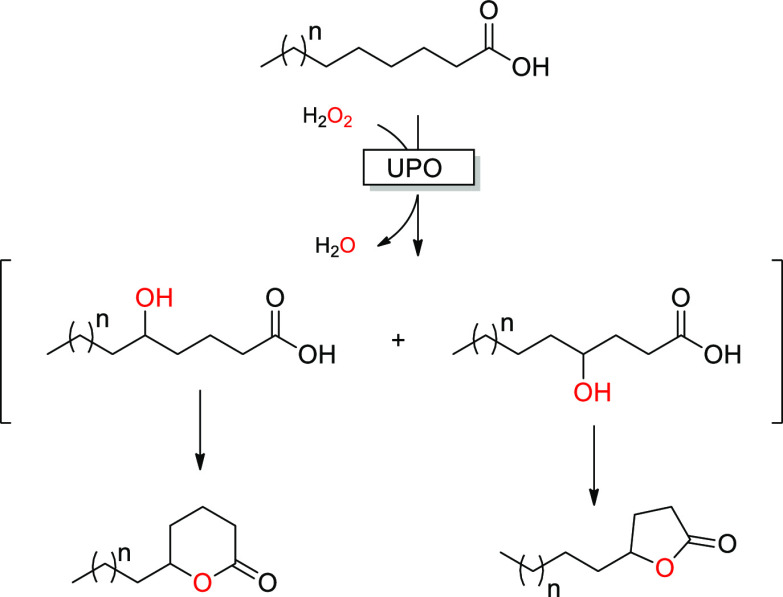
UPO-Catalyzed Lactonization of Saturated Fatty Acids

**Figure 1:**
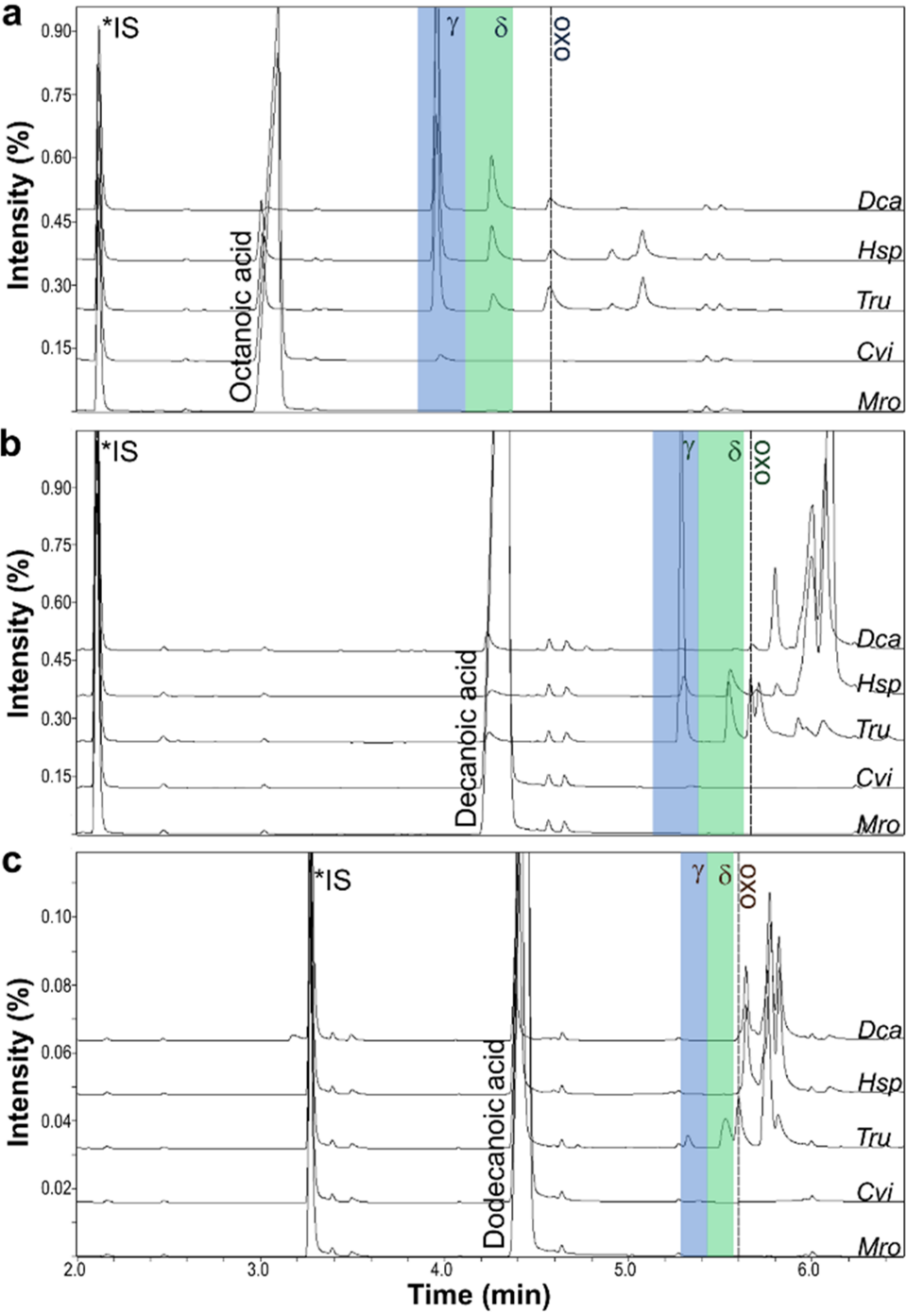
GC-MS analysis of UPO-catalyzed hydroxylation and lactonization of (a) octanoic acid, (b) decanoic acid, and (c) dodecanoic acid. γ- and δ-lactone peaks are highlighted in blue and green, respectively. Overoxidation to the oxo acids is indicated by dashed lines. Reaction conditions: 200 mM KP_i_ buffer (pH 7.0), 20 μM UPO, 0.2 unit mL^–1^ GOx, 100 mM glucose, 10 mM fatty acid, 1% (v/v) acetone, 25 °C, 24 h, and shaking at 200 rpm.

Near complete conversion of decanoic acid was also observed with the same three UPOs, but with only *Tru*UPO yielding significant amounts of the lactones (Figure 1b). The same was true for dodecanoic acid (Figure 1c), with only *Tru*UPO yielding lactone but at significantly lower levels and only ∼70% conversion.

The samples were silylated to separate the individual hydroxylation products and determine the regioselectivity of each UPO (Table 1, Table S1, and Figures S4 and S5). Of the UPOs, *Dca*UPO showed the highest regioselectivity for the C4 and C5 positions of octanoic acid (95% of total products), with only traces of 2,- 3-, 6-, and 7-hydroxy fatty acids observed. *Hsp*UPO and *Tru*UPO showed ∼60–80% selectivity for the C4 and C5 positions of octanoic acid. The regioselectivity of *Tru*UPO was shifted more toward both the carboxyl and methyl terminal ends, giving the highest concentration of 3- and 7-hydroxyoctanoic acid of the UPOs.

**Table 1:** Regioselectivities Obtained for Fatty Acid Biotransformations with UPOs^a^

		product distribution (%)										
substrate	enzyme	C2	C3	C4	C5	C6	C7	C8	C9	C10	C11	diols
octanoic acid	*Dca*UPO	<1	3	78	17	1	1					
	*Hsp*UPO	1	8	61	17	3	10					
	*Tru*UPO	1	20	47	14	3	14					
decanoic acid	*Dca*UPO	–	1	5	7	10	25	23	29			
	*Hsp*UPO	–	8	2	5	9	19	23	34			
	*Tru*UPO	<1	12	48	21	4	5	4	6			
dodecanoic acid	*Dca*UPO	–	–	–	–	–	<1	16	38	22	16	8
	*Hsp*UPO	–	–	–	–	–	13	16	24	30	14	3
	*Tru*UPO	–	–	9	9	14	30	9	9	5	7	8

a Values are totals of hydroxy and oxo acid; values for C4 and C5 also include lactone. Reaction conditions: 200 mM KP_i_ (pH 7.0), 20 μM UPO, 10 mM fatty acid, 0.2 unit mL^-1^ of GOx, 100 mM glucose, 1% (v/v) acetone, 25 °C, shaking at 200 rpm, and 24 h.

All three UPOs showed a preference for C4 (γ-lactone) over C5 hydroxylation (δ-lactone) of ∼3.5–4.5-fold with octanoic acid. Increasing the fatty acid chain length to C10 shifted the regioselectivity of *Dca*UPO and *Hsp*UPO in favor of the subterminal positions (ω-1 to ω-3) with only small amounts of lactones formed (<10% of the total products). Only *Tru*UPO retained its selectivity for the C4 and C5 positions (48% and 21% of the total products formed, respectively). Similar to that of decanoic acid, the regioselectivity of *Dca*UPO and *Hsp*UPO was shifted to the subterminal positions on dodecanoic acid, with no lactone formation detected. The regioselectivity of *Tru*UPO was also decreased significantly, with almost all of the possible hydroxy fatty acids observed and the regioisomer ratios for C4 and C5 being <10% each. The three UPOs also displayed significant overhydroxylation of dodecanoic acid through the formation of diols, whereby the fatty acids were sequentially hydroxylated at more than one position.

The stereoselectivity of the UPOs in forming the major γ-lactones from C8 and C10 fatty acids was analyzed using chiral separation (Figure S7). *Dca*UPO, *Hsp*UPO, and *Tru*UPO yielded the same enantiomer from octanoic acid, with ee’s ranging between 84% and 93% (Table S6). *Tru*UPO was less enantioselective with decanoic acid, with the γ-lactone produced with an ee of 70%.

In an attempt to minimize the overoxidation of the C4 and C5 hydroxy fatty acids, which decreased the direct yield of the γ- and δ-lactones, the UPO concentrations were decreased to 10 μM and time-course analysis was performed (Figure 2). Despite the reactions not reaching full conversion, the C4 and C5 oxo acids already formed and were even present in small amounts in the initial stages of the reaction. Although the reaction rates decreased after ∼6 h, the lactone and hydroxy acids yields decreased relative to the total yields of the products formed. After 24 h, the oxo acids represented 10% of the C4- and C5-hydroxylated products with *Dca*UPO conversion of octanoic acid and 19% with *Tru*UPO conversion of decanoic acid.

**Figure 2:**
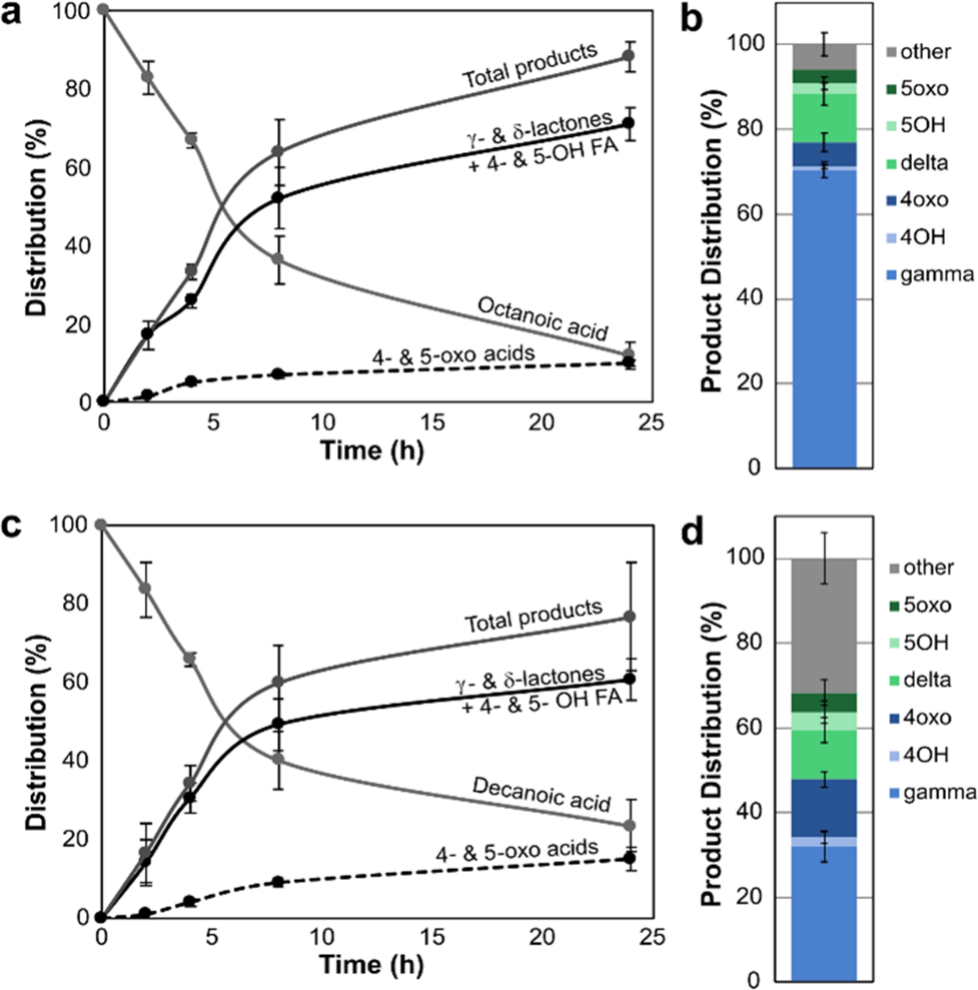
Time course of the conversion of (a) octanoic acid by *Dca*UPO and (C) decanoic acid by *Tru*UPO. (b and d) Stacked bar graphs show the formation of lactones compared to the total products obtained after 24 h. Reaction conditions: 200 mM KP_i_ buffer (pH 7.0), 10 μM UPO, 0.2 unit mL^–1^ GOx, 100 mM glucose, 10 mM fatty acid, 1% (v/v) acetone, 25 °C, and shaking at 200 rpm.

Recently, the overoxidation displayed by UPOs was advantageously used in the formation of chiral phenylethanols from ethylbenzene derivates. The UPO from *Agrocybe aegerita* (*Aae*UPO) was used in a bienzymatic cascade reaction, whereby the starting material was completely converted to the ketone and subsequently reduced to the alcohol using an alcohol dehydrogenase (ADH).^29^ Similarly, when the starting materials are racemic alcohols, chiral propargylic alcohols and amines can be formed via the UPO-catalyzed ketone followed by either an (*S*)- and (*R*)-selective ADH or an (*R*)-selective amine transaminase, respectively.^30^ We therefore attempted the same strategy with octanoic acid as a proof of concept, using *Dca*UPO and the ADH from *Micrococcus luteus* (*Ml*ADH).^31^ An ADH from *M*. *luteus* has been implicated in the natural synthesis of γ-dodecalactone from oleic acid,^32^ and *Ml*ADH has also previously been used for the oxidation of various fatty alcohols to the corresponding keto acids.^33,34^ In our initial attempts at this sequential bienzymatic cascade reaction (Scheme 2), octanoic acid was reacted with *Dca*UPO for 48 h to ensure full conversion and an excess of the oxo acids. The reaction mixture’s pH was adjusted to 6; the mixture was supplemented with 50 μM *Ml*ADH, 0.1 mM NADH, and 5% (v/v) isopropanol for cofactor regeneration, and the reaction allowed to proceed for 24 h.

**Scheme 2:**
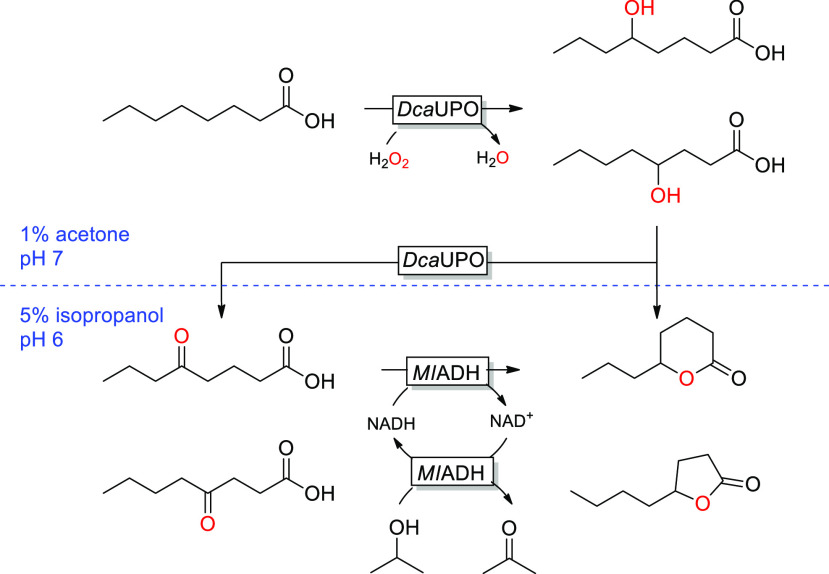
UPO and ADH Bienzymatic Cascade for the Conversion of Fatty Acids to γ- and δ-Lactones via an Oxo Acid Intermediate

After the first stage of the reaction, no γ-lactone was observed, with only small amounts of δ-lactone and the oxo acids constituting the major products. Despite the *Ml*ADH converting the oxo acids back to their hydroxy fatty acids, with subsequent lactonization, the final lactone yields were disappointingly low. Most likely, a futile cycle comprising *Dca*UPO-catalyzed alcohol oxidation and *Ml*ADH-catalyzed reduction accounted for this low yield. We therefore performed the reaction as a two-pot two-step cascade extracting the reactions after the initial *Dca*UPO-step dissolution in isopropanol and reconstitution with the *Ml*ADH and NADH system. Indeed, near complete conversion of the oxo acids to the γ- and δ-lactones was realized (Figure 3). The same enantiomer of the γ-lactone was observed in the bienzymatic cascade (Figure S7c) with comparable enantioselectivity (ee of 87%).

**Figure 3:**
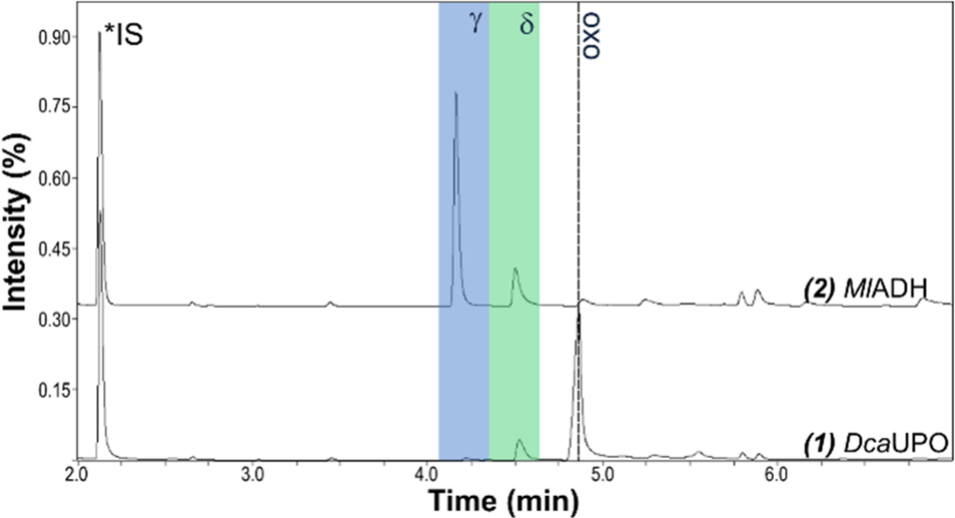
GC-MS analysis of the sequential bienzymatic cascade reaction after *Dca*UPO conversion of octanoic acid to C4 and C5 oxo acids (bottom) and their reduction to the corresponding C4 and C5 hydroxy fatty acids and subsequent lactonization to γ- and δ-lactones (lactone peaks highlighted in blue and green).

Finally, we scaled the reaction to 10 and 50 mL, yielding lactone concentrations of 0.4 and 0.5 g L^–1^, respectively. Complete conversion to the lactones was again observed, but with final product concentrations lower than expected, most likely due to the sequential extractions. Although the process is scalable, optimization of the extractions is therefore required. Alternatively, inactivation of UPO after complete conversion of the fatty acids can potentially resolve this. Also, spatiotemporal separation of both steps using a flow-chemistry approach appears to be a simple and economical solution.

Overall, we have demonstrated the ability of UPOs to selectively hydroxylate C8–C10 fatty acids at the C4 and C5 positions to yield the corresponding γ- and δ-lactones under mild reaction conditions using only H_2_O_2_. Moreover, high enantioselectivity was observed for the γ-lactones produced from C8 and C10 fatty acids. Admittedly, at present, we lack information about the absolute stereochemistry of the lactones from the UPO-catalyzed hydroxylation reactions. In future work, this will be addressed most likely through Mosher’s method using enantiomerically pure α-methoxy-α-trifluoromethylphenylacetic acid.^35,36^ We also envisage that the relatively low total turnover numbers [TTNs (Table S3)] can be improved, and the regioselectivity fine-tuned, through directed evolution for specific lactones. As with most UPO-catalyzed reactions, overoxidation of alcohols remains an obstacle. Although the extent of this overoxidation can be reduced through protein engineering of the UPO,^12^ a bienzymatic cascade reaction employing well-developed ADH systems for the reduction of the oxo acids resulting from overoxidation can be employed. Indeed, this system can be preferentially used as (*R*)- or (*S*)-selective ADHs will afford enantiopure lactones, irrespective of the enantiospecificity of the UPO employed.

## Data Availability

The data underlying this study are available in the published article and its Supporting Information.

## Supporting Information Available

Production and purification of biocatalysts, experimental setup, GC and GC-MS chromatograms, and GC-MS spectra of products (PDF)
