# Supplementary material for: Lactones from Unspecific Peroxygenase-Catalyzed In-Chain Hydroxylation of Saturated Fatty Acids
Source: Org Lett. 2023 Jun 30;25(27):4990–5. doi: 10.1021/acs.orglett.3c01601 (PMC10353034; doi:10.1021/acs.orglett.3c01601)
Supplement: Supplementary file 1 — ol3c01601_si_001.pdf [file ol3c01601_si_001.pdf]

Supporting Information

Lactones from unspecific peroxygenase catalyzed in-chain hydroxylation of saturated fatty acids

Ana C. Ebrecht[a], Thato M. Mofokeng[a], Frank Hollmann[b], Martha S. Smit[a], and Diederik J. Opperman[a]*

[a] Department of Microbiology and Biochemistry, University of the Free State, Bloemfontein, 9300, South Africa; [b] Department of Biotechnology, Delft University of Technology, Delft, 2629HZ, The Netherlands

Table of content

1. Cloning, expression and purification of biocatalysts

   Table S1. Unspecific peroxygenases (UPOs) used in study

   Figure S1. SDS-PAGE analysis and CO-difference spectra of purified UPOs and ADH

   Table S2. Purification yields of recombinant UPOs from *E. coli*

2. Biotransformations

   Table S3. Total turnover numbers (TTN) and Turnover frequency (TOF) for UPOs

   Figure S2. Time-course biotransformations

   Figure and Table S4. Product distribution

   Table S5. GC-FID and GC-MS methods

   Figure S4. GC-MS chromatograms and spectra data for reactions with octanoic acid

   Figure S5. GC-MS chromatograms and spectra data for reactions with decanoic acid

   Figure S6. GC-MS chromatograms and spectra data for reactions with dodecanoic acid

   Figure S7. Chiral separation of γ-lactones

   Table S6. Enantioselectivity (*ee* values) for γ-lactones

1. Cloning, expression, and purification of biocatalysts

The genes encoding unspecific peroxygenases (UPOs, Table S1) were codon optimized for expression in *Escherichia coli* and synthesized by GeneScript (USA). The open reading frames were cloned into the expression vector pET28a(+), via *Nde*I and *Bam*HI.

Table S1: Unspecific peroxygenases (UPOs) used in study

Biocatalysts	Organism	NCBI/PDB Accession No
*Dca*UPO	*Daldinia caldariorum*	KAI1463234.1
*Hsp*UPO	*Hypoxylon sp.* EC38	OTA57433.1
*Tru*UPO	*Talaromyces rugulosus*	QKX61976.1
*Mro*UPO	*Marasmius rotula*	7ZBP (PDB)
*Cvi*UPO	*Collariella virescens*	7ZCL (PDB)

The constructs were transformed into *Escherichia coli* BL21Gold-(DE3) and heterologous expression of the UPOs were performed in auto-induction media (ZYP-5052)[1] supplemented with 0.5 mM δ-aminolevulinic acid hydrochloride and 50 μM $FeCl_3 \cdot 6H_2O$ (16°C for 96 h, 200 rpm).

For purification, cells were harvested by centrifugation (7000 x g, 10 min, 4°C) and resuspended (0.2 g wet weight mL^{-1}) in buffer A (25 mM Tris-HCl pH 8.0) containing 1% (v/v) Triton X-100. Disruption of the cells was carried out by single passage through a One-Shot Cell disrupter System (Constant Systems Ltd) at 30 kPsi, followed by centrifugation (30 000 x *g*, 40 min, 4°C). The resulting soluble fraction was loaded onto a 1 mL His GraviTrap™ column (Cytiva), previously equilibrated with buffer B (25 mM Tris-HCl, 300 mM NaCl, 40 mM imidazole, pH 8). The loaded column was washed with 10 column volumes of buffer B. Protein was eluted in buffer E (25 mM Tris-HCl, 300 mM NaCl, 500 mM imidazole, pH 8.0), and desalted using PD-10 columns (GE Healthcare) equilibrated with buffer D (10 mM potassium phosphate buffer, 150 mM NaCl pH 7.4).

Micrococcus luteus alcohol dehydrogenase (*Ml*ADH) were expressed from pET28b(+) and purified as previously described.[2]

Glucose oxidase (GOx) from *Aspergillus niger* was purchased from Sigma-Aldrich.

Protein concentration was determined by Pierce BCA assay (ThermoFisher Scientific) using bovine serum albumin as a standard. Active UPO were confirmed using CO-difference spectra.

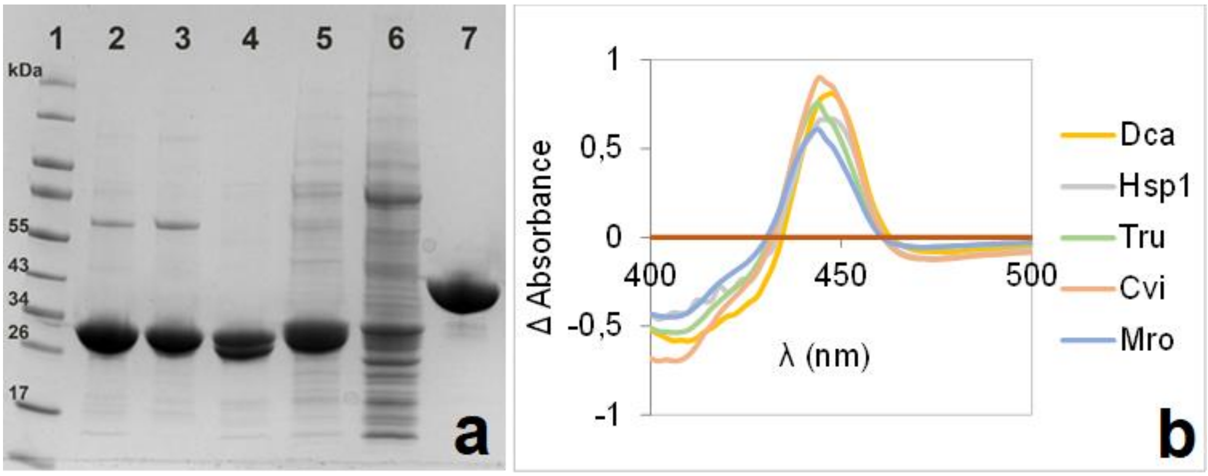

Figure S1. Purification of unspecific peroxygenases and alcohol dehydrogenase. (a) SDS-PAGE analysis of purified UPOs. Lane1: Molecular weight marker, lane 2: *Dca*UPO, lane 3: *Hsp*UPO, lane 4: *Tru*UPO, lane 5: *Cvi*UPO, lane 6: *Mro*UPO, lane 7: *Ml*ADH. **(b)** CO-difference spectra measured after setting up biotransformation.

Table S2: Purification yields of recombinant UPOs from *E. coli*

Biocatalysts	Protein Conc.
*Dca*UPO	15.8 mg L^{-1}
*Hsp*UPO	24.3 mg L^{-1}
*Tru*UPO	10 mg L^{-1}
*Mro*UPO	3.5 mg L^{-1}
*Cvi*UPO	24.1 mg L^{-1}

2. Biotransformations

Biotransformations were performed in 2 mL microcentrifuge tubes, with a reaction mixture consisting of 200 mM potassium phosphate buffer (pH 7.0), 100 mM glucose, 10-20 µM UPO, 1% (v/v) acetone, 0.2 U mL^{-1} GOx in a final volume of 1 mL. Reactions were started with the addition of 10 mM of fatty acid and incubated at 25 °C for 2, 4, 8, and 24 h with shaking (200 rpm).

Reactions were stopped and extracted by the addition of 150 µL HCl (5 M), followed by 1 mL ethyl acetate containing 2 mM internal standard (3-octanol or 1-undecanol). Samples were analysed by GC-FID (Shimadzu GC-2010) and GC-MS (Thermo Scientific TraceGC ultra – Trace DSQ) using the columns and temperature programs described in Table S2.

For better separation, samples were silylated. For silylation, 100 µL of the ethyl acetate extracts from the biotransformations were dried under N_2 at room temperature. N,O-Bis(trimethylsilyl)acetamide containing 2% (w/v) trimethylchlorosilane were added to the dried samples (50 µL), mixed and incubated at 70°C for 3 h and analyzed by GC-FID and GC-MS. Product concentrations were calculated using commercial standards of γ-octalactone, γ-decalactone, and γ-dodecalactone for lactones formed, and 8-hydroxy octanoic acid, 10-hydroxy decanoic acid, and 12-hydroxy dodecanoic acid for hydroxy fatty acids formed. The remaining substrate concentration was calculated using their corresponding standards.

Table S3. Total turnover numbers (TTN) and Turnover frequency (TOF) for UPOs

	**TTN** [$\mu mol_{product}/\mu mol_{UPO}$]		
Substrate	*Octanoic acid*	*Decanoic acid*	*Dodecanoic acid*
*Dca*UPO	1120	870	770
*Hsp*UPO	790	933	670
*Tru*UPO	750	800	760
	**TOF** [min^{-1}]		
*Dca*UPO	0.9	1.0	0.6
*Hsp*UPO	0.7	1.8	0.3
*Tru*UPO	0.6	0.9	0.6

*TTN achieved over 24 h reactions. TTN were calculated for the total product formed after 24 h ([UPO]= 10 µM). TOF were calculated for 4 h reactions ([UPO]= 10 µM).

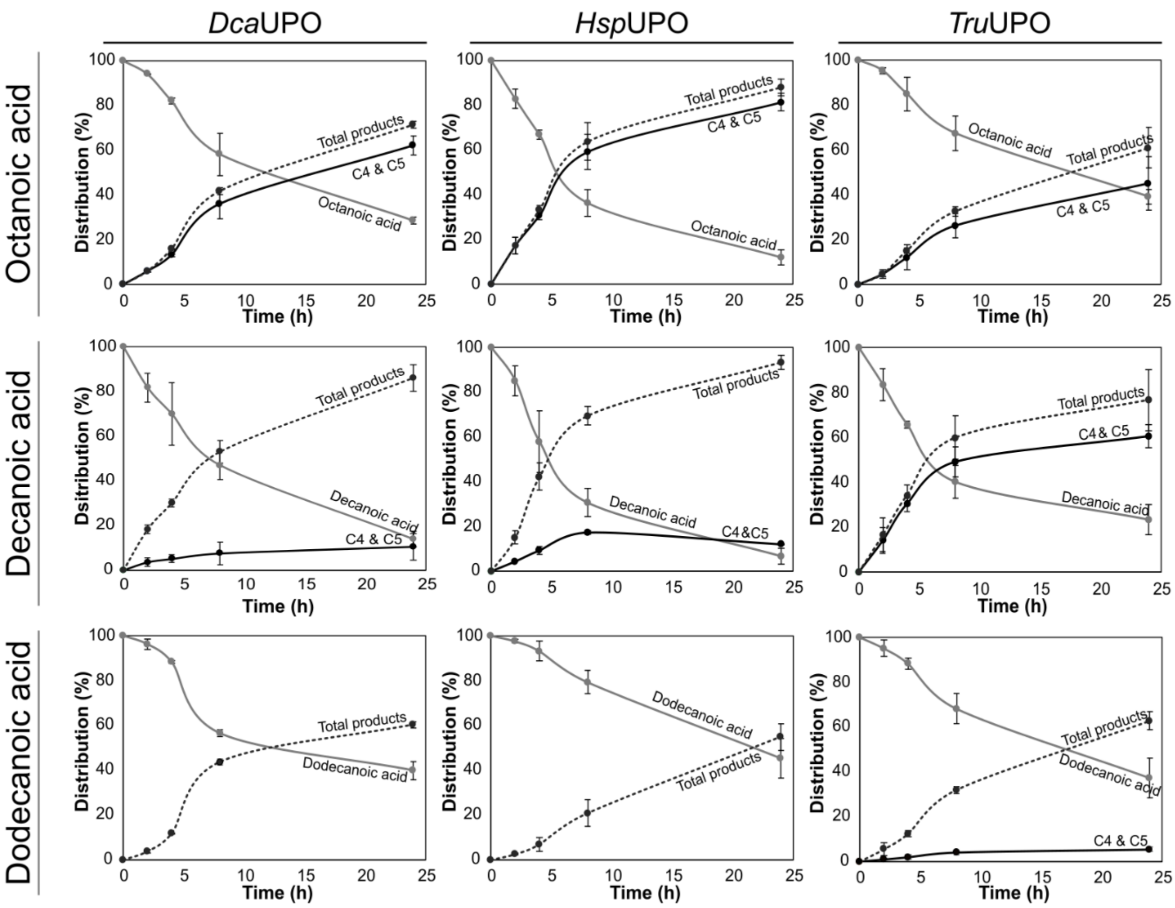

Fig S2. Time course reactions for the formation of lactones and hydroxy fatty acids by the different UPOs. Plots show decrease of substrate (grey), formation of hydroxy- and keto-acids in positions C4 and C5 (C4 & C5, black), and total product formed at the different time points (Total products, broken lines), for octanoic acid (first row), decanoic acid (second row), and dodecanoic acid (third row). Columns indicate biotransfromations for *Dca*UPO (first column), *Hsp*UPO (second column), and *Tru*UPO (third column).

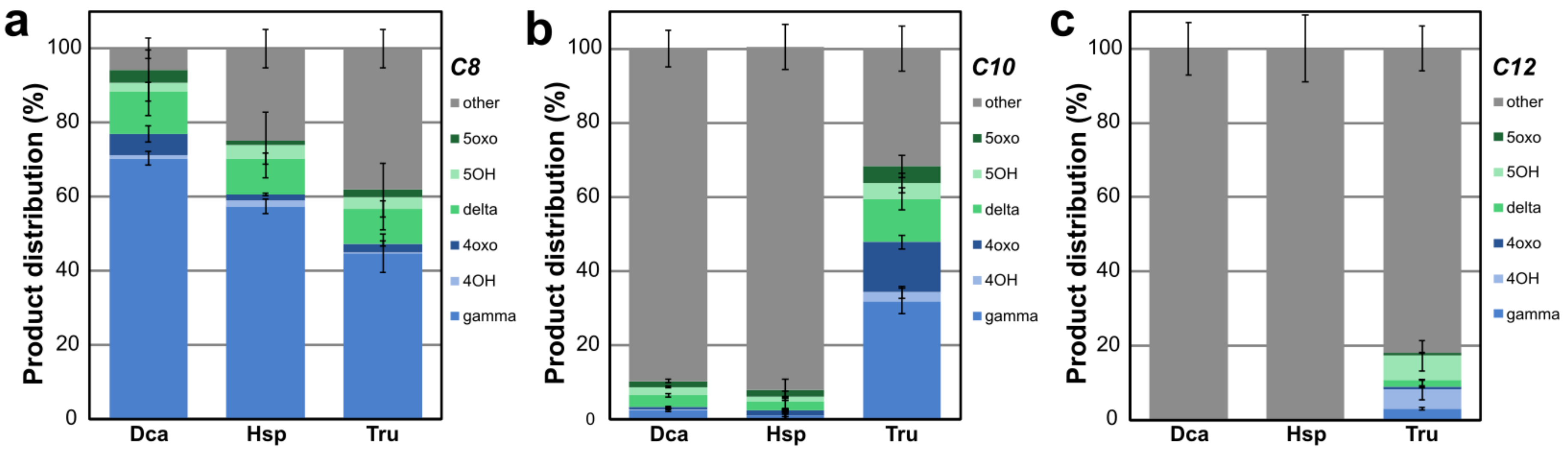

Fig S3. Lactones and hydroxy fatty acids produced by the different UPOs. Percentage of product distribution was calculated for 24 h biotransformations with **(a)** octanoic acid, **(b)** decanoic acid, and **(c)** dodecanoic acid. Other (grey) indicate formation of hydroxy- and keto-acids in positions different from C4 (blue) and C5 (green).

Table S4. Percentage of products obtained for biotransformations with *Dca*, *Hsp*, and *Tru*UPO.

Substrate	Enzyme	Product distribution (%)										
		C2	C3	C4	C5	C6	C7	C8	C9	C10	C11	Diols
Octanoic acid	*Dca*UPO	<1	3	78(71/1/6)	17(12/2/3)	1	1					
	*Hsp*UPO	1	8	61(57/1/3)	17(11/4/2)	3	10					
	*Tru*UPO	2	20	47(45/<1/2)	14(9/3/2)	3	14					
Decanoic acid	*Dca*UPO		1	5(3/<1/2)	7(3/2/2)	10(9/1)	25(18/7)	23(19/4)	29(25/4)			
	*Hsp*UPO		8	2(1/<1/1)	5(2/1/2)	9(8/1)	19(18/1)	23(22/1)	34(33/1)			
	*Tru*UPO	<1	12	48(32/2/14)	21(12/4/5)	4(2/2)	5(5/<1)	4(4/<1)	6(6/<1)			
Dodecanoic acid	*Dca*UPO		-	-	-	-	<1	16(14/2)	38(34/4)	22(20/2)	16(16/<1)	8
	*Hsp*UPO		-	-	-	-	13(7/6)	16(13/3)	24(21/3)	30(16/14)	14(8/6)	3
	*Tru*UPO		-	9(3/5/1)	9(2/6/1)	14(13/1)	30(26/4)	9(8/1)	9(8/1)	5(4/1)	7(5/2)	8

Values in brackets indicate percentage of hydroxy-acid/keto-acid; for oxyfunctionalyzation of C4 and C5 is indicated percentage of lactone/hydroxy-form/oxoacid. Reaction conditions: [potassium phosphate] = 200 mM pH 7.0, [UPO] = 20 uM, [fatty acid] = 10 mM, [GOX] = 0.2 U, [glucose]= 100 mM, [acetone] = 1% (v/v), T = 25 °C, shaking = 200 rpm, t = 24 h.

Table S5. GC-FID and GC-MS methods.

Substrate	Derivatization	Program	Column
Octanoic acid<br>*Decanoic acid*<br>*Dodecanoic acid*	BSTFA	100 °C hold 1 min → 200 °C (5 min^{-1}) hold 5 min<br>100 °C hold 1 min → 300 °C (10 min^{-1}) hold 5 min	FactorFour VF-5ms column (60 m x 0.32 mm x 0.25 µm, Varian)
Octanoic acid<br>*Decanoic acid*<br>*Dodecanoic acid*	none	120 °C hold 1 min → 200 °C (8 min^{-1}) hold 5 min	FactorFour VF-5ms column (60 m x 0.32 mm x 0.25 µm, Varian)
Chiral analysis of γ-lactones	none	40 °C hold 5 min → 150 °C (5 min^{-1}) hold 20 min	CHIRALDEX B-TA column (30 m × 0.25 mm x 0.12 µm)

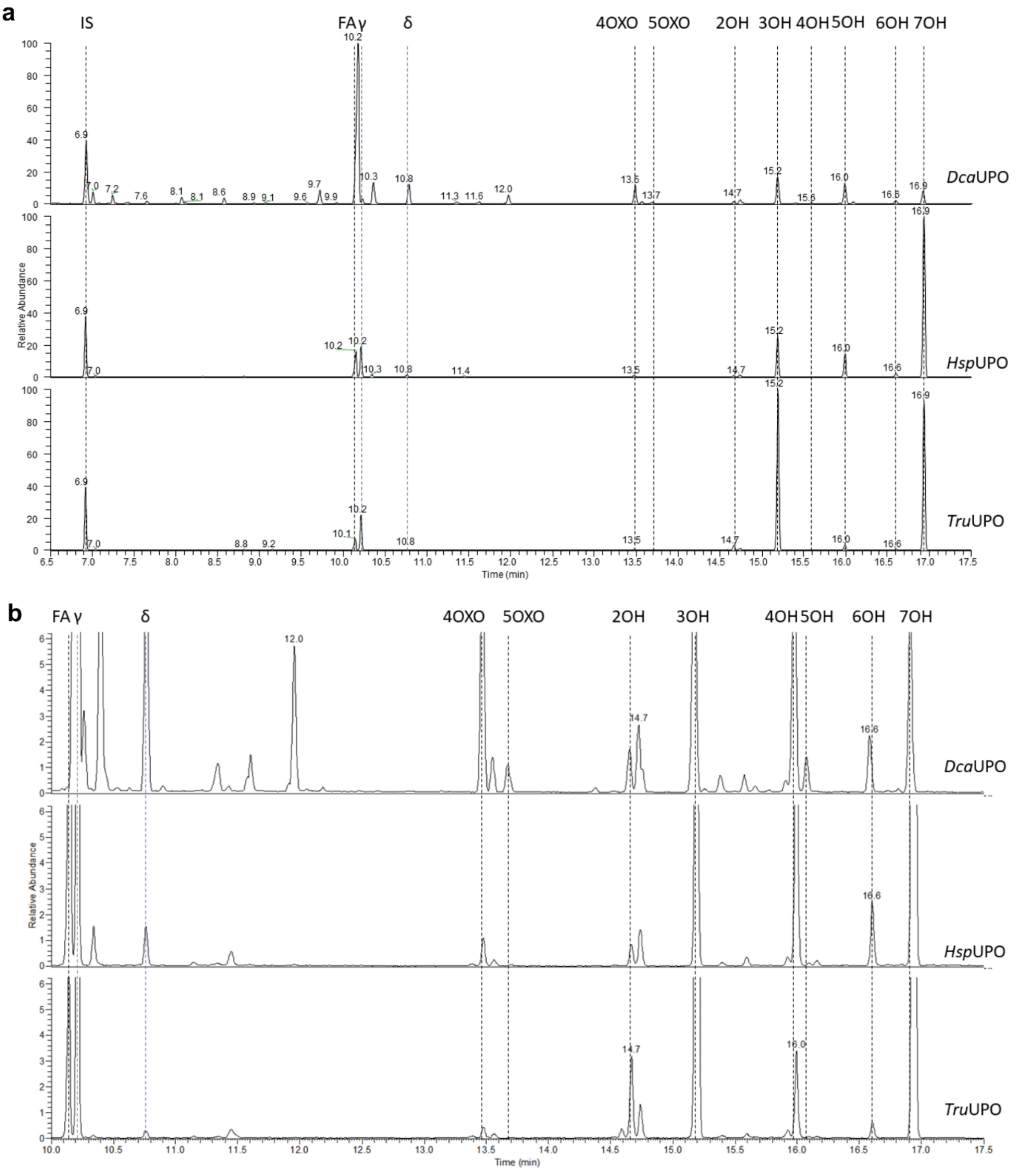

Fig S4. Identification of product formed for reactions with octanoic acid. (a) GC-MS chromatograms of syllilated samples for biotransformations with *Dca*UPO*, Hsp*UPO*,* and *Tru*UPO. **(b)** Zoom into the product peaks of the chromatograms. **(c)** Spectra of products obtained by oxyfunctionalization of position C4 and in position C5. **(d)** Spectra of hydroxy- fatty acids in positions different from C4 and C5.

c

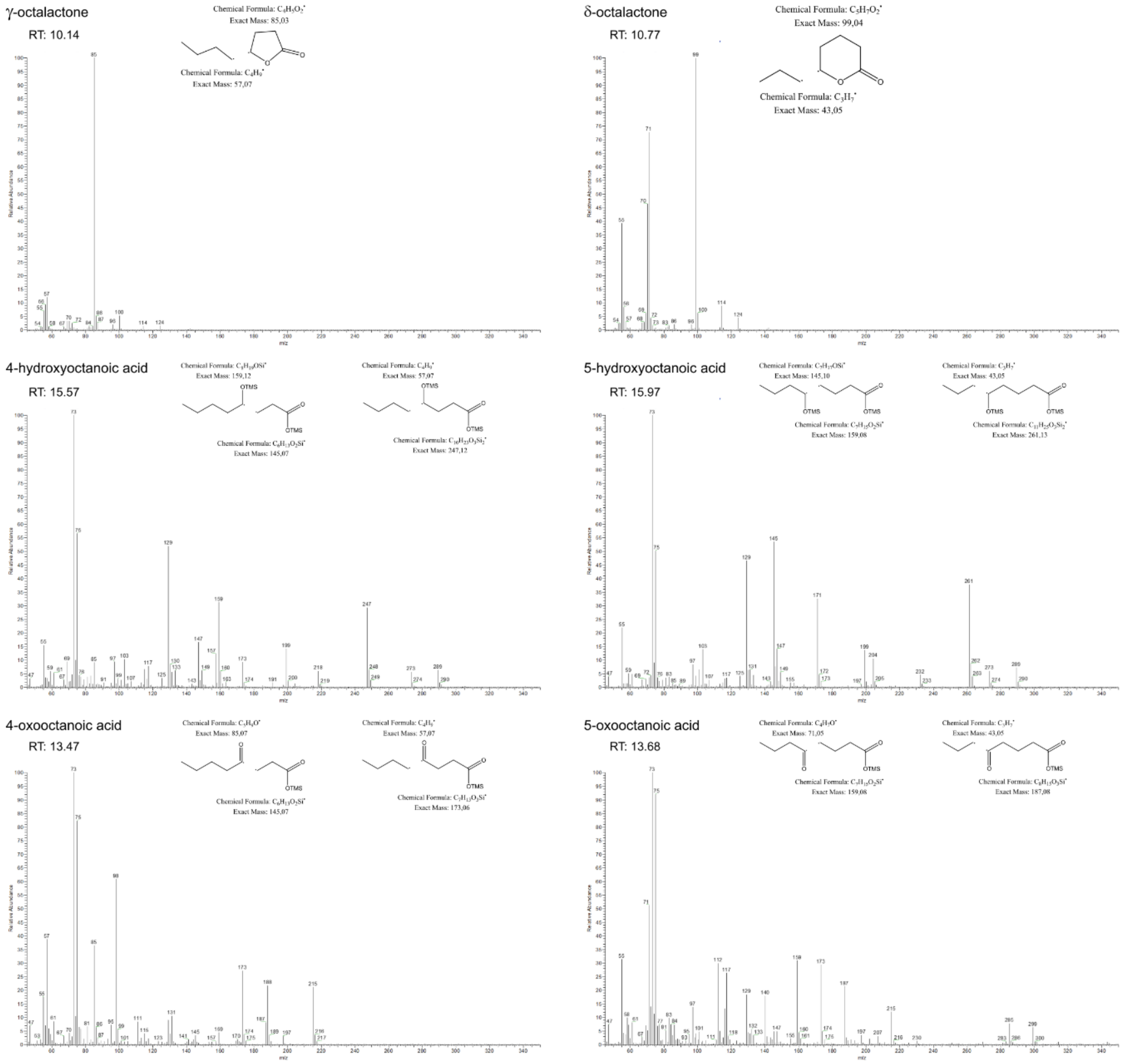

d

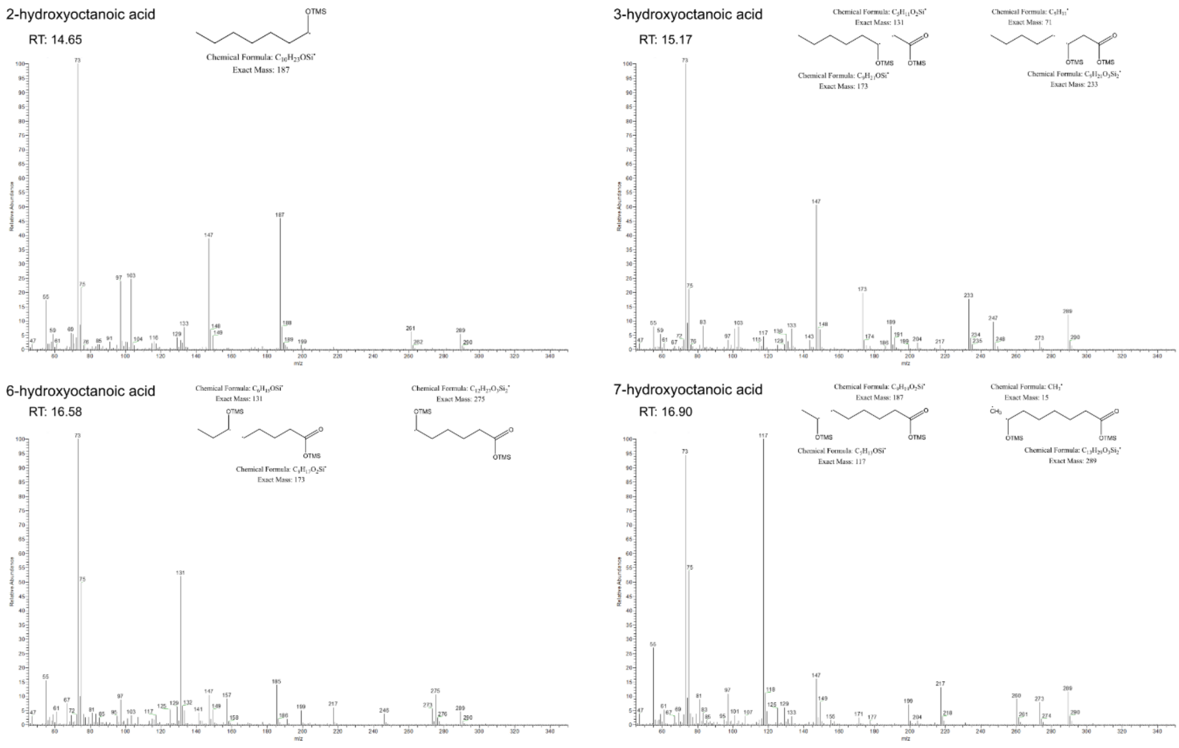

Fig S4. cont.

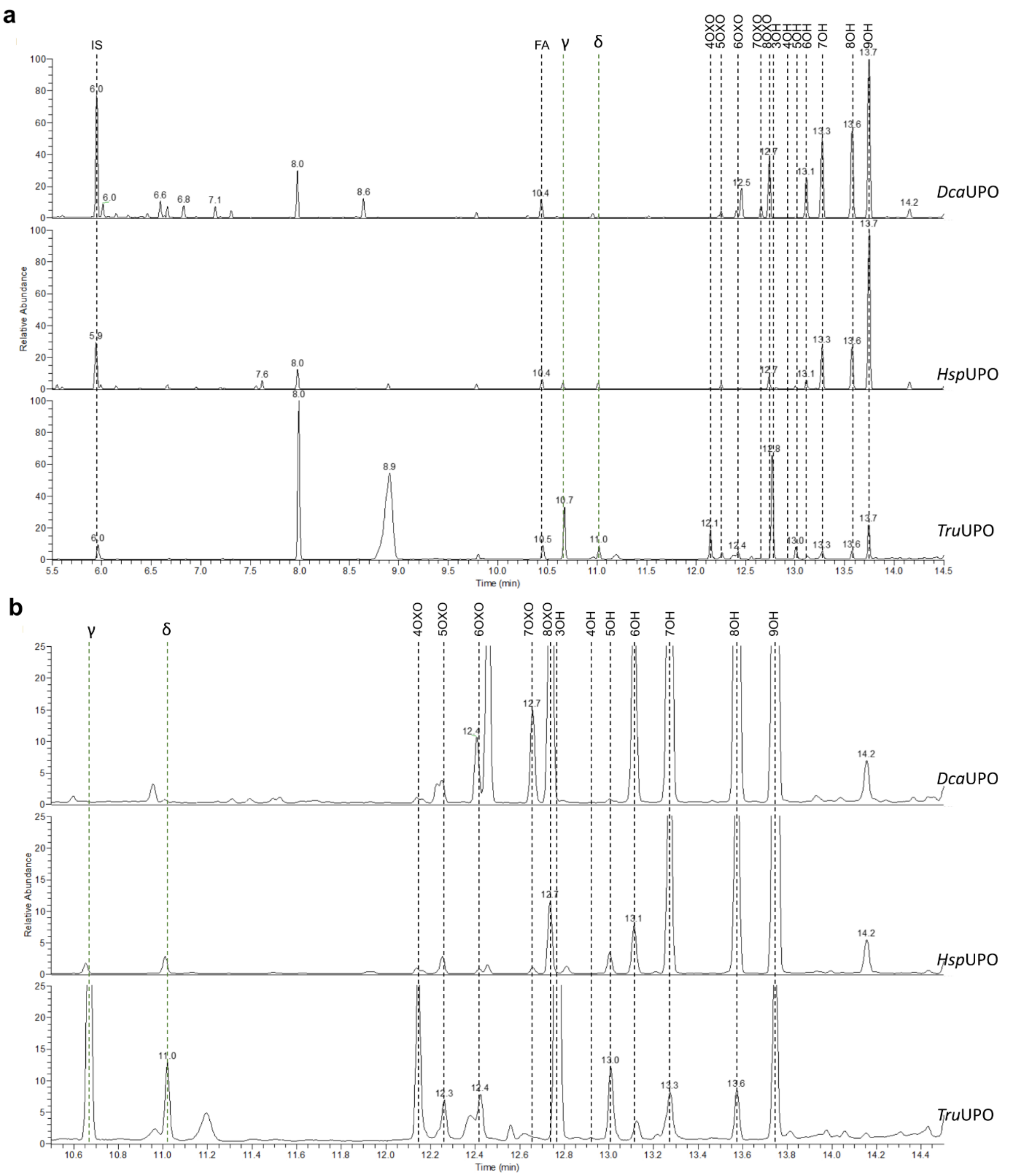

Fig S5. Identification of product formed for reactions with decanoic acid. (a) GC-MS chromatograms of syllilated samples for biotransformations with *Dca*UPO*, Hsp*UPO*,* and *Tru*UPO. **(b)** Zoom into the product peaks of the chromatograms. **(c)** Spectra of products obtained by oxyfunctionalization of position C4 and in position C5. **(d)** Spectra of keto- and hydroxy- fatty acids in positions different from C4 and C5.

c

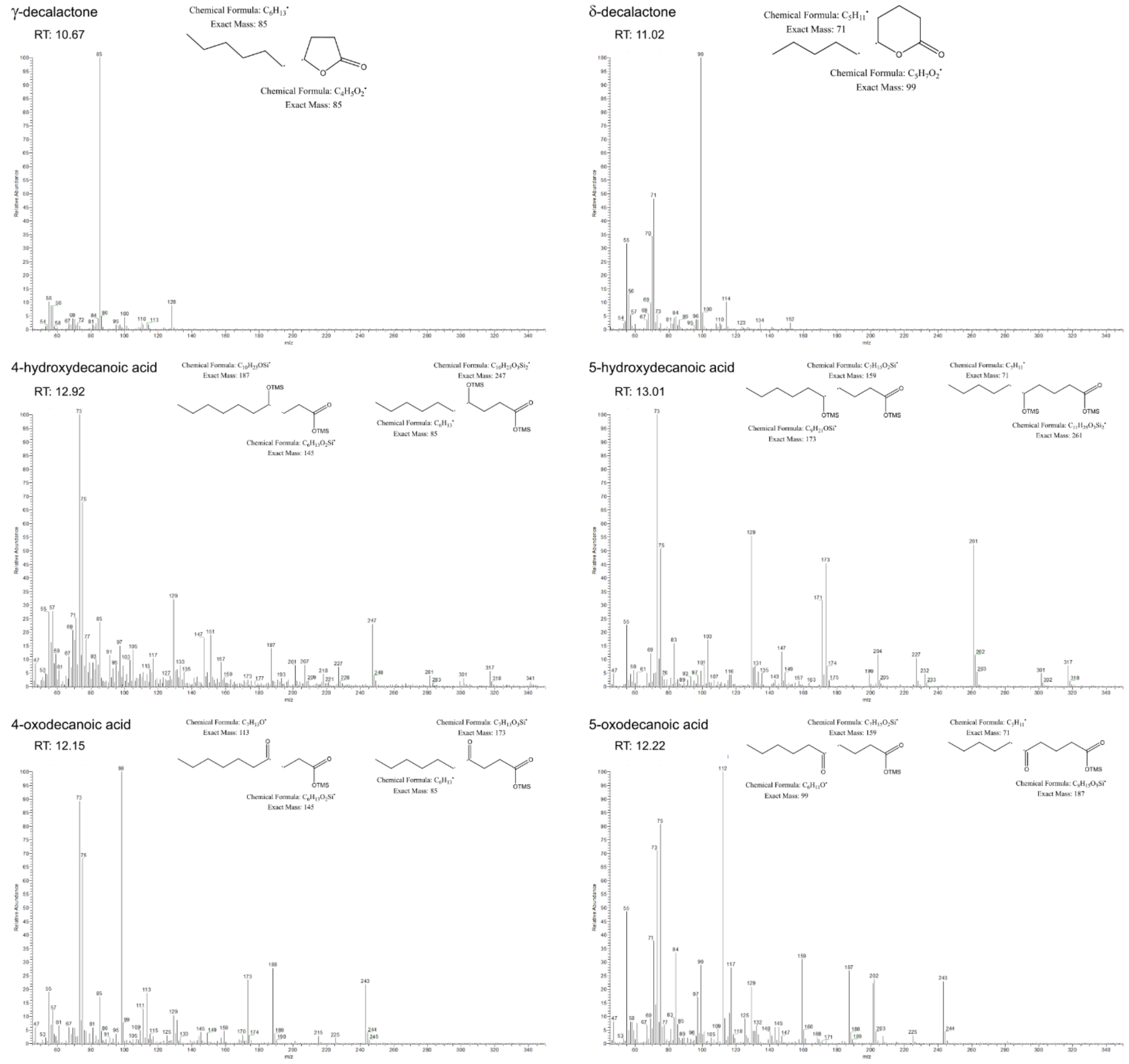
γ-decalactone
RT: 10.67
δ-decalactone
RT: 11.02
4-hydroxydecanoic acid
RT: 12.92
5-hydroxydecanoic acid
RT: 13.01
4-oxodecanoic acid
RT: 12.15
5-oxodecanoic acid
RT: 12.22

Fig S5. cont.

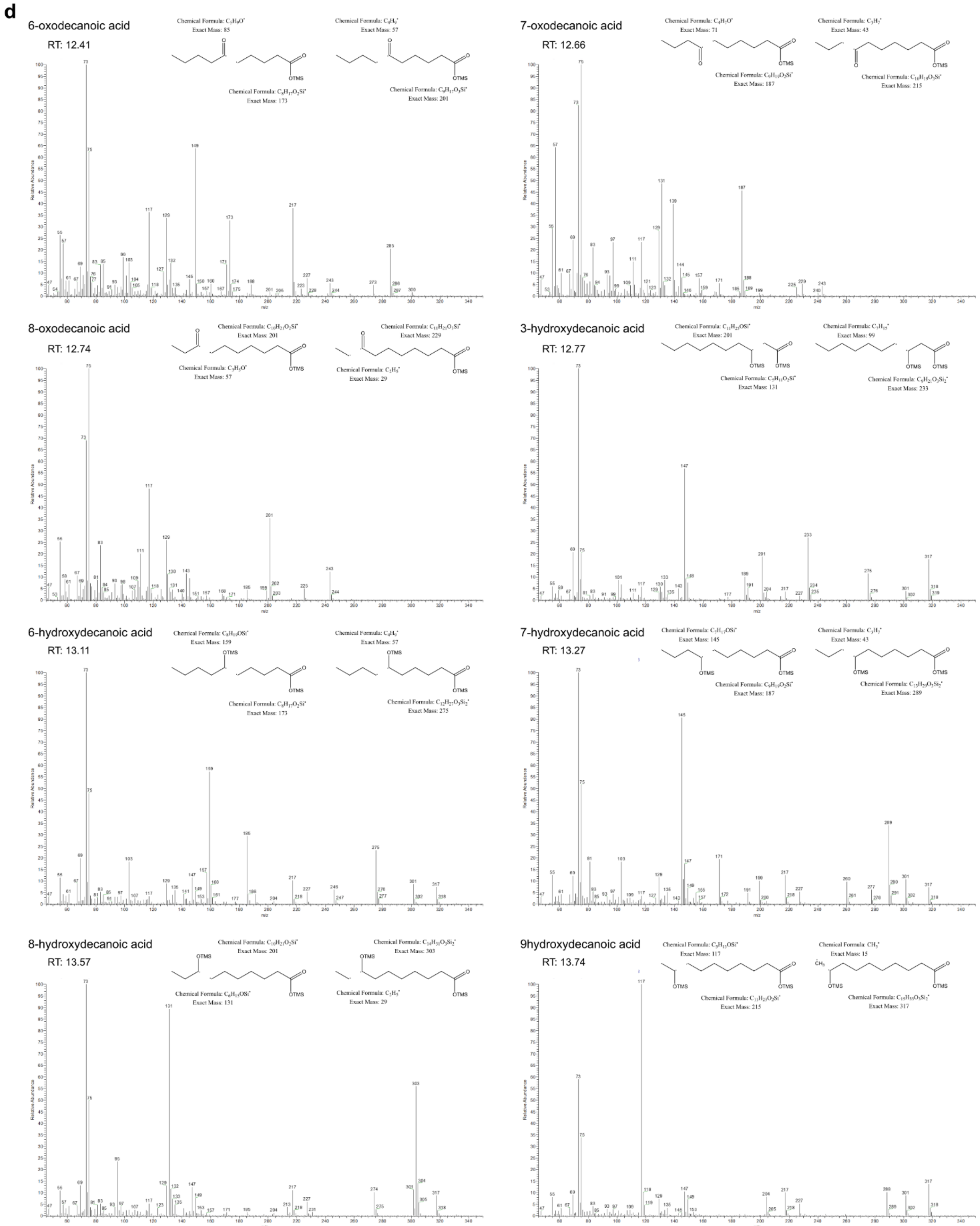
d
6-oxodecanoic acid
RT: 12.41
7-oxodecanoic acid
RT: 12.66
8-oxodecanoic acid
RT: 12.74
3-hydroxydecanoic acid
RT: 12.77
6-hydroxydecanoic acid
RT: 13.11
7-hydroxydecanoic acid
RT: 13.27
8-hydroxydecanoic acid
RT: 13.57
9hydroxydecanoic acid
RT: 13.74

Fig 5. cont.

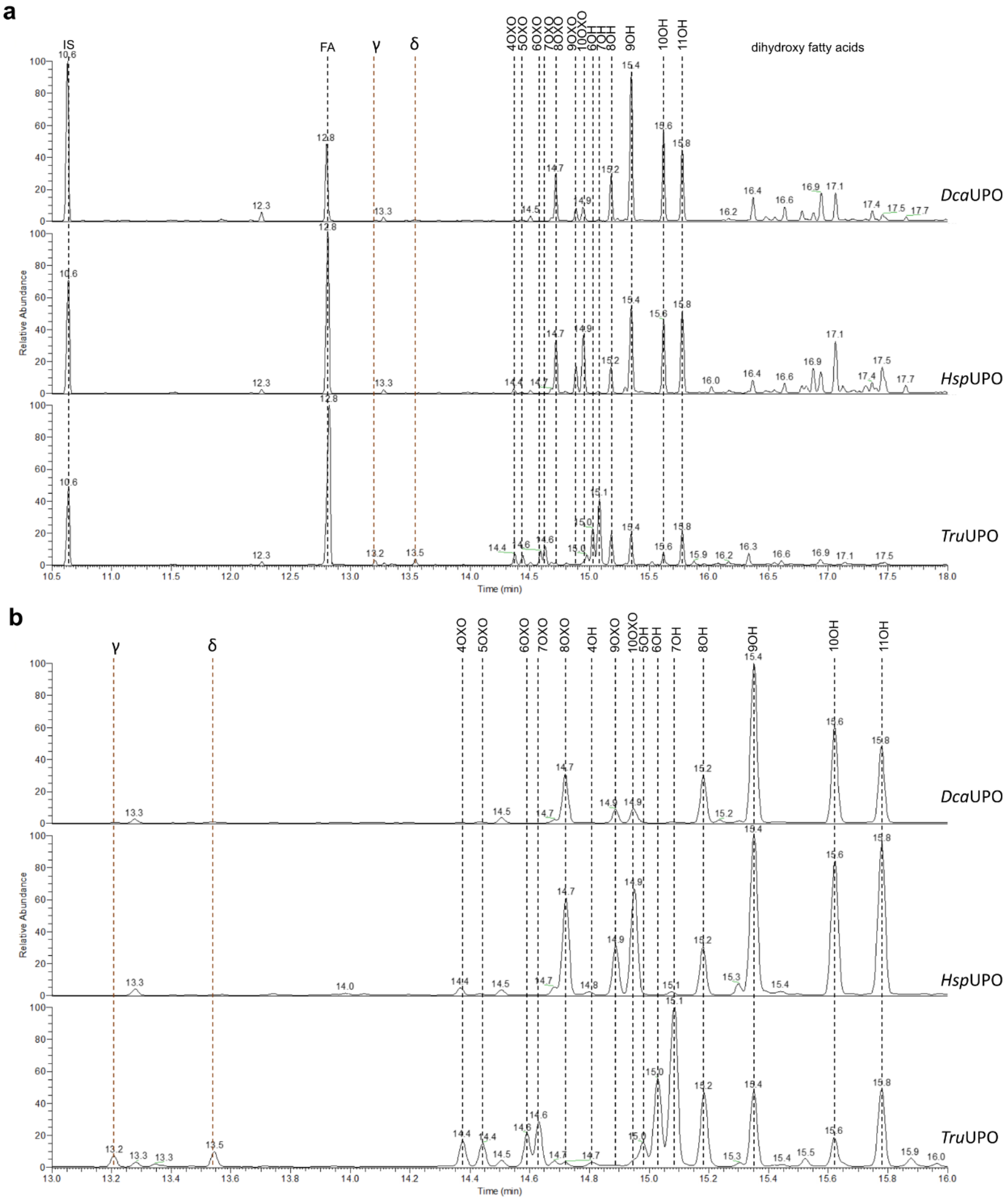

Fig S6. Identification of product formed for reactions with dodecanoic acid. (a) GC-MS chromatograms of syllilated samples for biotransformations with *Dca*UPO*, Hsp*UPO*,* and *Tru*UPO. **(b)** Zoom into the product peaks of the chromatograms. **(c)** Spectra of products obtained by oxyfunctionalization of position C4 and in position C5. **(d)** Spectra of keto-fatty acids in positions different from C4 and C5. **(e)** Spectra of hydroxy-fatty acids in positions different from C4 and C5

c

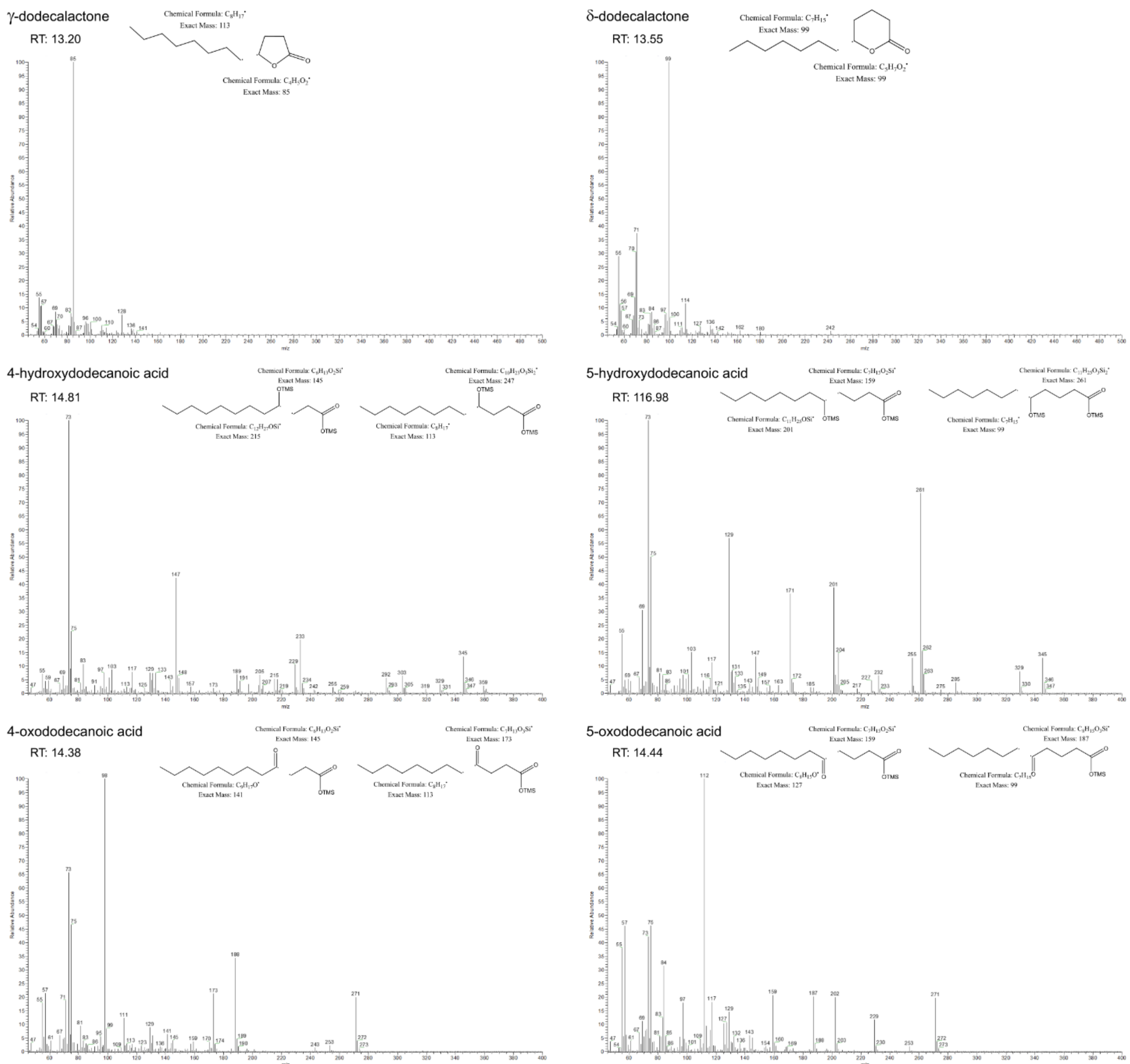

γ-dodecalactone
RT: 13.20
δ-dodecalactone
RT: 13.55
4-hydroxydodecanoic acid
RT: 14.81
5-hydroxydodecanoic acid
RT: 116.98
4-oxododecanoic acid
RT: 14.38
5-oxododecanoic acid
RT: 14.44
Relative Abundance
m/z
OTMS

Fig S6. cont.

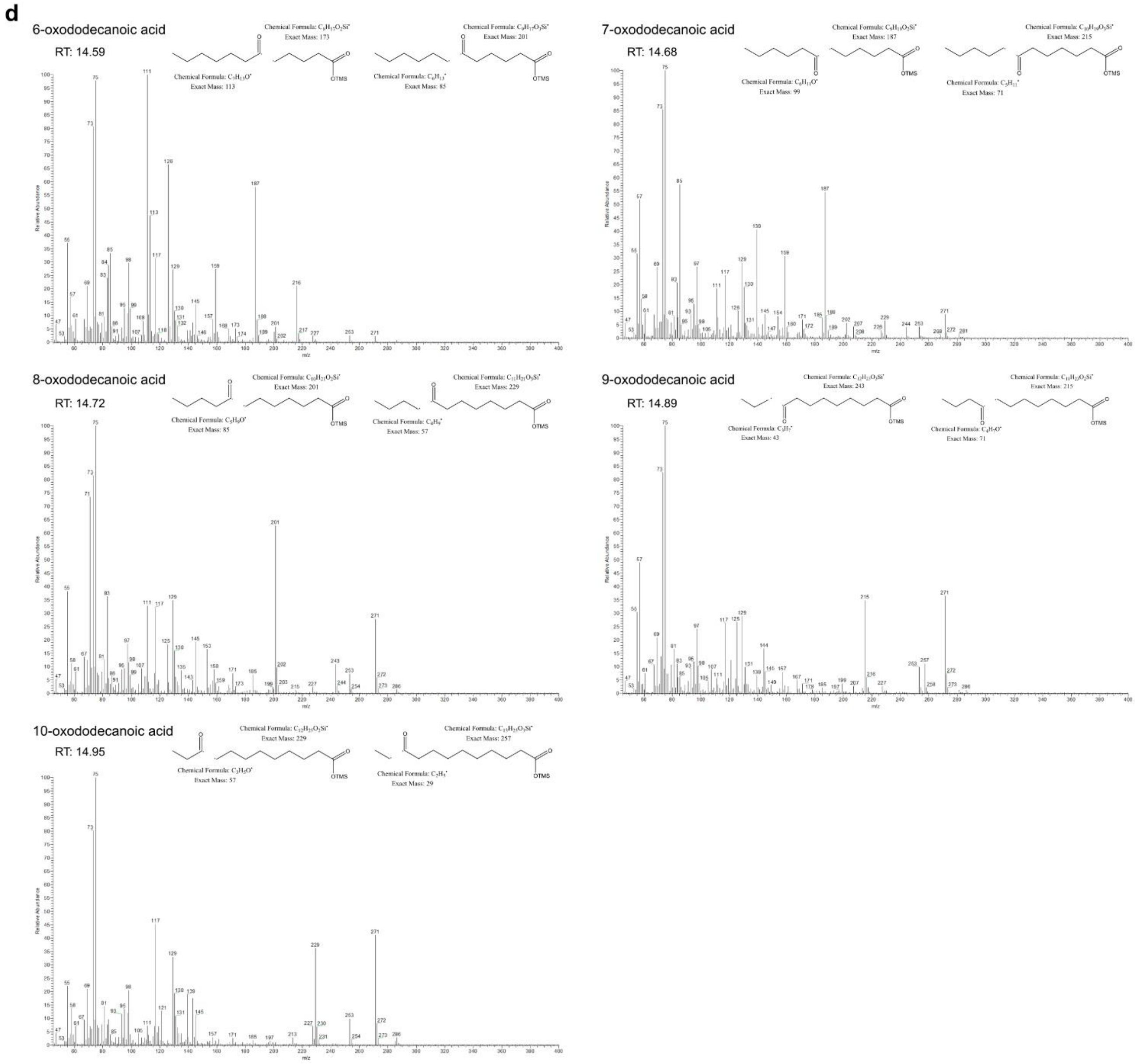

d
6-oxododecanoic acid
RT: 14.59
7-oxododecanoic acid
RT: 14.68
8-oxododecanoic acid
RT: 14.72
9-oxododecanoic acid
RT: 14.89
10-oxododecanoic acid
RT: 14.95

Fig S6. cont.

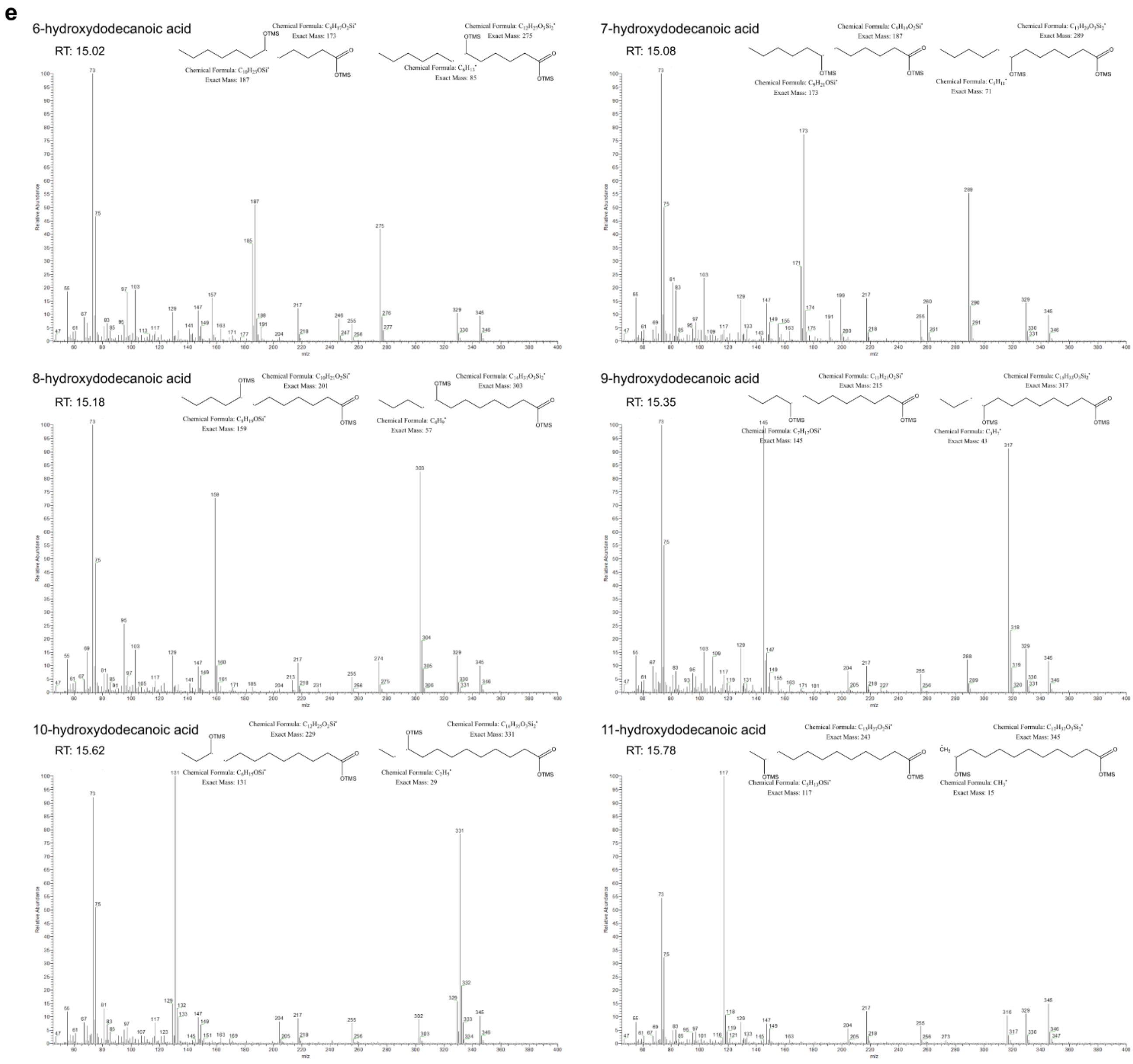
e
6-hydroxydodecanoic acid
RT: 15.02
7-hydroxydodecanoic acid
RT: 15.08
8-hydroxydodecanoic acid
RT: 15.18
9-hydroxydodecanoic acid
RT: 15.35
10-hydroxydodecanoic acid
RT: 15.62
11-hydroxydodecanoic acid
RT: 15.78

Fig S6. cont.

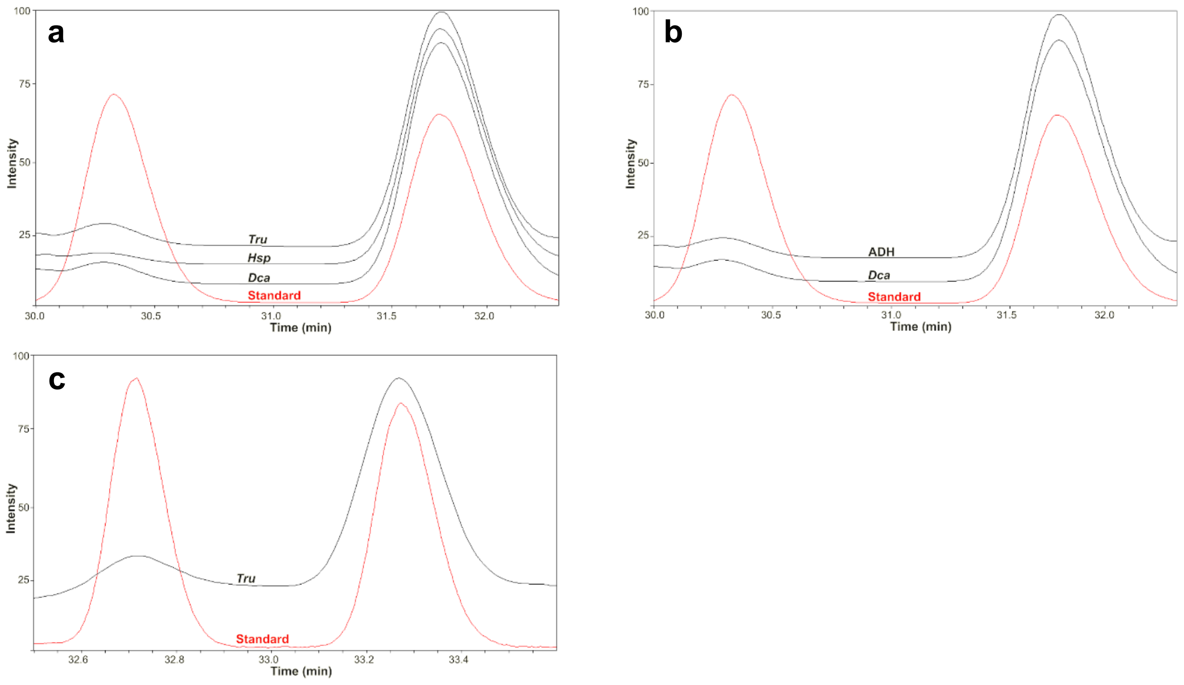

Fig S7. Chiral analysis of γ-lactones. a) Chromatograms of γ-octalactone produced by *Dca*UPO, *Hsp*UPO, and *Tru*UPO. Standard (racemic) of γ-octalactone is shown in red. **b)** Chromatograms of γ-octalactone produced by *Dca*UPO, and *Ml*ADH. **c)** Chromatograms of γ-decalactone produced by *Tru*UPO. Standard (racemic) of γ-decalactone is shown in red.

Table S6. Enantioselectivity of UPOs for γ-lactones.

**UPO**	**Product**	***ee.* (%)**
Dca + ADH	γ-octalactone	87
Dca	γ-octalactone	88
Hsp	γ-octalactone	93
Tru	γ-octalactone	84
Tru	γ-decalactone	70
